# A rare presentation of triple‐barrel aortic dissection in a Ghanaian male

**DOI:** 10.1002/ccr3.8708

**Published:** 2024-04-02

**Authors:** Nana Adjoa M. Coleman, Aba A. Folson, Mawufemor A. Bekui, Bennett Owusu

**Affiliations:** ^1^ Department of Internal Medicine Ho Teaching Hospital Ho Ghana; ^2^ Department of Internal Medicine and Therapeutics, School of Medicine University of Health and Allied Sciences Ho Ghana

**Keywords:** aortic, dissection, Ghanaian, triple‐barrel

## Abstract

Acute aortic dissection in unusual/rare circumstances may become chronic and multi lumen. A high clinical acumen, examination and investigations in the acute setting, may help reduce late/misdiagnosis and complications in low resource settings.

## 1 INTRODUCTION

Aortic dissection is the most common of the acute aortic syndromes (70%–80% of cases).^1^ The other two are intramural hematoma and penetrating aortic ulcers. It often occurs acutely and is usually catastrophic.^2^ It occurs when there is a tear in the intimal lining of the aorta, creating an “intimal flap” and in effect, two or more lumen in the arterial wall (false lumen). Twenty percent of all cases die before reaching the hospital, and in those who survive, mortality rates increase by 1%–2% per hour without treatment.^3^ Diagnosis requires a high index of suspicion as its presentation may mimic other conditions. A study by Kurabayashi et al determined the rate of misdiagnosis of aortic dissection on initial assessment in all patients presenting to the emergency room with chest pain as 16%.^4^ While relatively uncommon (13 cases reported in the English literature),^5^ a few cases may be asymptomatic.

Multiple barreled aortic dissections, with a new lumen being created in the false lumen, or an acute dissection occurring in the setting of a chronic dissection are very rare with a few cases described in the literature. We present a case of a delayed diagnosis of extensive triple‐barreled aortic dissection with limited peripheral arterial vascular involvement.

## 2 CASE REPORT

### 2.1 History and examination

A 63‐year‐old male was referred to the cardiology clinic with a 9‐month history of recurrent central chest pain that radiated to his back. He had been diagnosed with hypertension 3 years prior and was compliant with medication.

The chest pain that had occurred 9 months earlier was sharp and described as tearing and sometimes burning, radiating to his back (interscapular region), neck, and left flank. The initial episode was graded 10/10 and was associated with dyspnoea and lightheadedness leading to a 5‐day hospital admission. Thereafter, he sought medical care outpatient basis from various facilities on account of non‐specific chest pain and was eventually referred to our facility for further assessment and management.

In the initial review with our facility, he admitted to palpitations and visual disturbances but denied easy fatigue, syncope, unilateral weakness, nausea, diaphoresis, lower limb pain or paraesthesia, abdominal pain or discomfort or pedal swelling. There was no claudication.

He was of average height and reported both parents and siblings to be of average height and build. He had no history of hyper‐mobile joints or frequent joint dislocations, and neither did any of his siblings. There was no family history of cardiac illness or sudden cardiac death.

On assessment at the cardiac clinic, he was hemodynamically stable, not obese (BMI: 19.2 kg/m^2^) and in no acute distress. Vital signs were as follows: blood pressure 144/100 mmHg in the right arm and 147/94 mmHg in the left arm, pulse rate 70 beats/min, respiration rate 18/min, temperature 36.2°C, and oxygen saturation of 99% on room air. The cardiac exam showed radio‐radial synchrony, and all other pulses were equal and synchronous. The cardiac apex was in the sixth left intercostal space in the mid‐clavicular line with an apical heave and a thrill. There was a loud second heart sound best heard in the aortic area with a grade 2 ejection systolic murmur loudest at the aortic area which radiated to the back. Lung bases were clear on auscultation. There was no clinical evidence of limb ischaemia. All other systems were grossly normal.

### 2.2 Investigation and treatment

Initial laboratory investigations, which included a full blood count, renal function test and liver function test were unremarkable.

**FIGURE 1:**
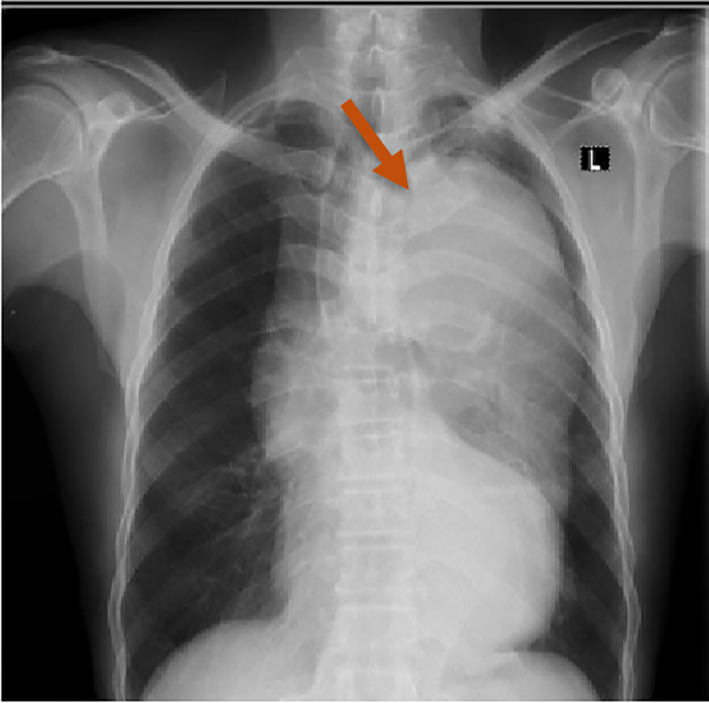
Chest radiograph showing widened mediastinum and prominent aortic shadowing.

A chest x‐ray revealed a widened mediastinum with a left‐sided soft tissue mass that had displaced the trachea to the contralateral side, which heightened suspicion of an aortic dissection (Figure 1).

**FIGURE 2:**
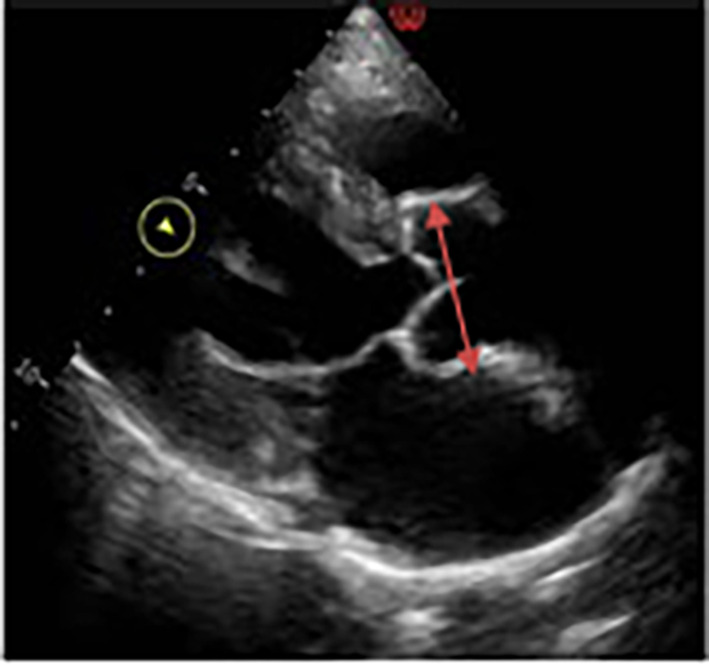
2D echocardiogram showing non‐involvement of the aortic root and part of the ascending aorta.

The ECG showed voltage criterion left ventricular hypertrophy with no evidence of coronary artery disease. On 2D echocardiography, the aortic root and ascending aorta appeared normal in size with no intimal flap noted. The suprasternal view was suboptimal and no evidence of a dissection was seen at that time. There was no associated aortic regurgitation however mild concentric hypertrophy of the left ventricle with normal systolic function and moderate dilatation of the left atrium were noted. The right ventricle appeared normal in size with no evidence of pulmonary hypertension seen (Figure 2).

**FIGURE 3:**
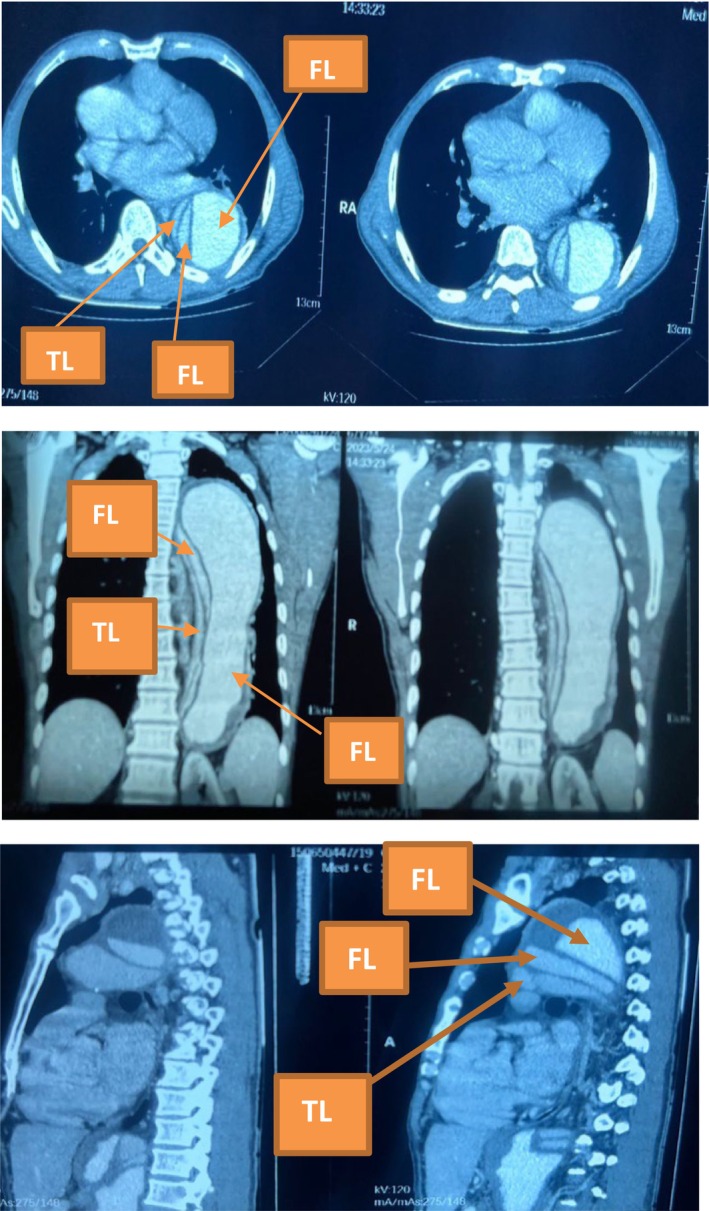
Contrast CT scan of the chest showing triple lumen aortic dissection.

A contrast computerized tomography scan of the chest, obtained on the day he presented, showed a triple‐barreled aortic dissection involving the aortic arch and the descending thoracic aorta, throughout its course and down to the celiac trunk. There was one true lumen, two false lumens and two distinct intimal flaps. The true lumen was continuous with the aortic root superiorly and the celiac trunk inferiorly. The ascending aorta was normal. The true lumen had a diameter of 0.5 cm and the larger of the false lumens was 4.0 cm whilst the other was 0.8 cm. The ascending aorta was normal (Figure 3).

### 2.3 Outcome and follow‐up

The need for possible surgical correction was discussed with the patient and cardiac surgeons but declined with the patient opting for medical therapy as he was concerned about the cost of cardiac surgery. Medical therapy was initiated with beta‐blockers and an angiotensin receptor blocker. BP has been maintained at less than 120/70 mmHg and an average pulse rate of 60 beats/min. The patient has had no complaints and is stable on periodic follow‐up reviews.

## 3 DISCUSSION

The incidence of aortic dissection in the United States is reported to be 5–30 cases per 1 million people per year and out of every 1000 patients presenting to the emergency department with acute back, chest, or abdominal pain, three of them are found to have an acute aortic dissection.^6^ Similar data in Africa is lacking as factors such as the availability of advanced imaging systems and high mortality rate influence the confirmation of diagnosis.

Risk factors for aortic dissection include advanced age, hypertension, connective tissue disorders, pre‐existing aortic aneurysms, bicuspid aortic valves, coarctation of the aorta, trauma and inflammatory diseases.^7^

Most cases present with sudden onset severe tearing or ripping chest (ascending aorta) and/or back pain (descending aorta) with intensity usually worse on onset, and which radiates anywhere between the thorax and abdomen. There may be associated dyspnoea and hyper‐ or hypotension on presentation, and signs of end‐organ ischaemia (i.e., focal neurologic deficits from cerebral ischaemia, abdominal organ ischaemia, lower limb ischaemia, etc.).^8^

Our patient had been managed for dyspepsia for months before his visit with us, due to the non‐specific nature of his symptoms, localization of pain to the left flank and lateral chest wall and apparent absence of signs of end‐organ damage or life‐threatening features. Aortic dissection is often missed as presentation, especially in females and in the elderly may be atypical and in rare cases asymptomatic.^9^

The fundamentals of an accurate diagnosis rest on having a high index of suspicion, comprehensive history taking and a thorough physical exam. A history of sudden onset, severe chest pain which may be central and radiating to the back or flank in a patient with a history of hypertension, atherosclerotic disease, connective tissue disorders or other genetic predisposition should make a clinician suspect a possible aortic dissection. Patients with aortic dissection usually present with hemodynamic instability culminating in circulatory collapse (usually from proximal dissection), or with hypertension (which is also a risk factor for aortic dissection) especially with distal dissection.^10^, ^11^ These classical findings were absent in our patient. Pulse deficits or absences occur in 50% of patients with proximal aortic dissection, the brachiocephalic artery being more commonly affected. All pulses should be examined to pick up absent pulses or pulse deficits.^11^

Risk scoring systems such as the Aortic dissection detection risk scoring (ADD‐RS) have been developed in conjunction with the d‐dimer to increase clinical suspicion and prompt further investigations in high‐risk patients who may present atypically.^10^ Serum D‐dimer levels are elevated in acute aortic dissection, according to a study on biomarkers by the International Registry of Acute Aortic Dissection (IRAD) and have high negative predictive value (>90%), hence can be used as an effective clinical marker in ruling out aortic dissection.^12^

**FIGURE 4:**
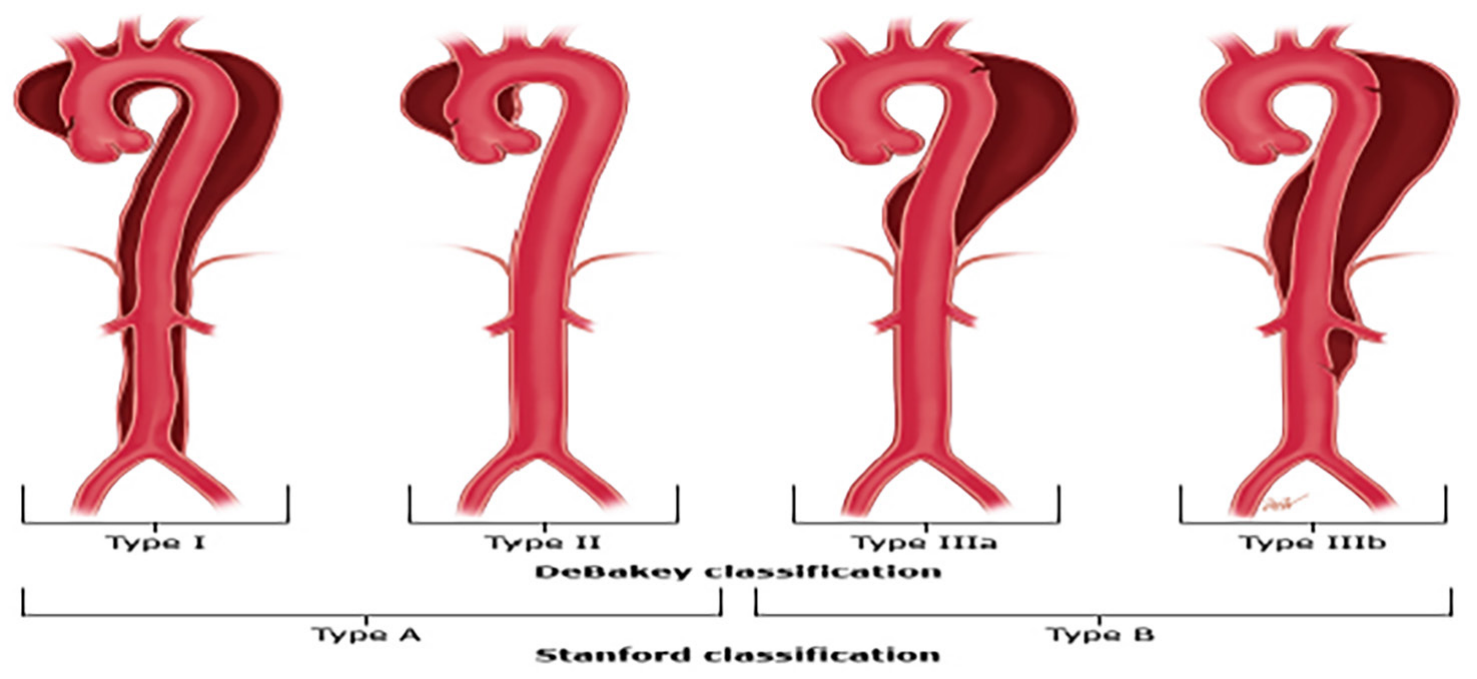
Stanford and Debakey classification of aortic dissection.

There are two systems of classification of aortic dissection based on anatomical location: The Stanford and DeBakey classifications^12^ (Figure 4).

Multiple‐barrelled aortic dissections (Triple‐barreled, quadruple‐barrelled) however are uncommon with a few cases described in literature due to the poor outcomes. They are usually of the Stanford type B morphology and occur in patients with Marfan's disease and other connective tissue diseases. In triple‐barrelled dissections, the new dissection may occur within the old, (i.e., True‐False‐False), or as a new dissection, markedly narrowing the true lumen (False‐True‐False) and causing significant symptoms due to end‐organ damage/ hypoperfusion. New onset chest pain in a patient previously diagnosed with aortic dissection should raise suspicion for a new dissection that is, multi‐barreled dissection.^13^

In a study done by Suyoeshi et al, multi‐barreled aortic dissection was found to be a powerful independent predictor of Aortic dissection‐related deaths.^14^ 75% of patients with multi‐barreled dissection (15/20) had AD‐related death over 140 months, as compared to 17.4% in the double‐barreled type.^15^

In the evaluation of patients with recurrent chest pain which seems non‐specific, simple outpatient radiographic tests such as a chest x‐ray, electrocardiogram and echocardiogram should be performed early to rule out uncommon but sinister causes which tend to portend a poor prognosis.^16^ The delay in diagnosis of this particular patient could be attributed to the fact that in our part of the world, such simple yet important investigative tools are not readily available in smaller healthcare facilities. Training and retraining of health workers and encouraging early referral to higher facilities and ensuring that these facilities have these basic infrastructure may help reduce late diagnosis.

Medical management is indicated as the modality of choice in uncomplicated descending aortic dissections (Stanford type B, DeBakey type III), estimated to be about 70% of all type B dissections.^17^ It is also instituted preoperatively, intra‐ and post‐operatively in patients being prepared for surgical correction. In acute type A aortic dissection with an unfavorable prognosis, as determined by risk scoring, medical management alone may be instituted.

The goal of medical management in aortic dissection is to reduce aortic wall stress due to the rate and velocity of ventricular contraction and blood pressure. Initial targets are a heart rate of 60 beats/min and systolic blood pressure between 100 and 120 mmHg.

Emergency surgical management is indicated for type A aortic dissections and complicated type B aortic dissection.^17^ The treatment approach traditionally involves open heart surgery. Endovascular surgery is increasingly used for descending thoracic aortic dissections (type B Aortic dissection). It is the recommended approach for complicated hyperacute or acute aortic dissections with favorable anatomy and prognosis. In multi‐lumen aortic dissection, optimal management involves open heart surgery with identification and resection of the initial intimal tear subsequently followed by grafting.

## 4 CONCLUSION

This case report has described chronic triple‐barreled aortic dissection with the main emphasis being to highlight the usefulness of good clinical acumen and basic investigations to diagnose this high‐risk condition in the acute setting. Though advanced diagnostics and surgical and or interventional modalities are largely inaccessible in Ghana, simple, accessible and useful tools such as a good history and physical exam, chest x‐ray, d dimer, ECG and echocardiography may prevent the catastrophic misdiagnosis of acute aortic dissection and hopefully, we will have more than luck on our side in reducing the high mortality in acute dissection and the debilitating morbidity of chronic dissection in our setting.

## AUTHOR CONTRIBUTIONS

**Nana Adjoa M. Coleman:** Conceptualization; data curation; investigation; writing – original draft. **Aba Folson:** Conceptualization; supervision; writing – review and editing. **Mawufemor A. Bekui:** Conceptualization; supervision; writing – review and editing. **Bennett Owusu:** Conceptualization; investigation; writing – review and editing.

## FUNDING INFORMATION

There was no funding for this work.

## CONFLICT OF INTEREST STATEMENT

The authors have no conflict of interest.

## CONSENT

Written informed consent was obtained from the patient to publish this report in accordance with the journal's patient consent policy. A copy of the written consent is available for review by the Editor‐in‐Chief of this journal.

## Data Availability

All data generated or analyzed during this study are included in this published article (and its additional information files).
